# Metabolic Syndrome and Periodontitis—From Shared Mechanisms to Interdisciplinary Care: A Narrative Review of Clinical Evidence

**DOI:** 10.3390/jcm15145510

**Published:** 2026-07-14

**Authors:** Anna-Maria-Clara Gherbon, Mirela Frandes, Deiana Roman, Adriana Gherbon, Luciana Maria Goguta, Romulus Timar, Oana Albai

**Affiliations:** 1 Doctoral School of Medicine, “Victor Babes” University of Medicine and Pharmacy, 300041 Timisoara, Romania; anna-maria-clara.gherbon@umft.ro; 2 Faculty of Bioengineering of Animal Resources, University of Life Sciences “King Mihai I” Timișoara, 300645 Timisoara, Romania; 3 Department of Functional Sciences—Medical Informatics and Biostatistics, “Victor Babes” University of Medicine and Pharmacy, 300041 Timisoara, Romania; 4 Center for Modeling Biological Systems and Data Analysis, “Victor Babes” University of Medicine and Pharmacy, 300041 Timisoara, Romania; 5 Department VII Internal Medicine—Diabetes, Nutrition, Metabolic Diseases, and Systemic Rheumatology, “Victor Babes” University of Medicine and Pharmacy, 300041 Timisoara, Romania; roman.deiana@umft.ro (D.R.); gherbon.adriana@umft.ro (A.G.); timar.romulus@umft.ro (R.T.); albai.oana@umft.ro (O.A.); 6 Railway Clinical Hospital, 300173 Timisoara, Romania; 7 Diabetes, Nutrition, and Metabolic Diseases, “Pius Brinzeu” Emergency Hospital, 300723 Timisoara, Romania; 8 Center of Molecular Research in Nephrology and Vascular Disease, “Victor Babes” University of Medicine and Pharmacy, 300041 Timisoara, Romania; 9 Department of Prosthodontics, Faculty of Dentistry, “Victor Babes” University of Medicine and Pharmacy, 300041 Timisoara, Romania; goguta.luciana@umft.ro; 10 Digital and Advanced Techniques for Endodontic, Restorative, and Prosthetic Treatment (TADERP) Research Center, “Victor Babes” University of Medicine and Pharmacy, 300041 Timisoara, Romania

**Keywords:** metabolic syndrome, periodontal disease, systemic inflammation, insulin resistance, oral microbiota, bidirectional relationship, periodontal therapy, cardiovascular disease, interdisciplinary care

## Abstract

**Background/Objectives**: Metabolic syndrome (MetS), defined by abdominal obesity, dysglycemia, dyslipidemia, hypertension, and insulin resistance, markedly increases the risk of type 2 diabetes mellitus and cardiovascular disease. Affecting an estimated 25–30% of the global adult population, MetS represents a major and growing public health challenge. A growing body of evidence supports a significant bidirectional relationship between MetS and oral health, particularly periodontitis. The present study aimed to synthesize current evidence on the pathophysiological mechanisms, epidemiological associations, interventional outcomes, and clinical implications of the bidirectional relationship between metabolic syndrome (MetS) and periodontitis. **Methods**: A narrative review following the SANRA framework was performed. PubMed, Scopus, and Web of Science were searched for articles published in January 2021–March 2026 using MeSH and free-text terms including “metabolic syndrome”, “periodontal disease”, “insulin resistance”, and “oral microbiota”. Eligible studies included original research and systematic reviews in English with full-text availability; animal and in vitro studies were included if directly informative of mechanistic pathways. **Results**: A total of 64 references were selected for inclusion. Shared mechanisms include chronic systemic inflammation, insulin resistance, oxidative stress, adipokine imbalance, endothelial dysfunction, and oral–gut microbiome dysbiosis. Cross-sectional and longitudinal studies show that MetS components are independently associated with higher prevalence and severity of periodontitis; meta-analyses report pooled odds ratios of 1.7–1.9 compared with metabolically healthy controls. Non-surgical periodontal therapy produces modest but significant reductions in glycated hemoglobin (HbA1c) and systemic inflammatory markers. Sodium–glucose cotransporter-2 (SGLT2) inhibitors may alter oral microbiota composition and cause mucosal changes, while glucagon-like peptide-1 (GLP-1) receptor agonists may increase caries risk through gastrointestinal side effects and xerostomia; both drug classes warrant proactive dental monitoring. **Conclusions**: The bidirectional relationship between MetS and oral health supports integrated screening and interdisciplinary management. Routine periodontal assessment should be integrated into the metabolic risk management pathway, and dental professionals should screen patients with severe periodontitis for metabolic risk factors. The oral microbiome emerges as a promising target for future mechanistic research and therapeutic intervention. Recognition of oral health as an integral component of metabolic health may improve risk stratification, prevention, and long-term patient outcomes. Large-scale randomized controlled trials with standardized endpoints are needed to establish causal directionality and optimize combined therapeutic strategies.

## 1. Introduction

Metabolic syndrome (MetS) is a cluster of interrelated cardiometabolic risk factors, including abdominal obesity, insulin resistance, dysglycemia, dyslipidemia, and hypertension, that together substantially increase the risk of type 2 diabetes mellitus (T2DM) and cardiovascular disease (CVD). Defined by the International Diabetes Federation (IDF) and harmonized joint criteria, MetS requires at least three of its five core components [1,2]. Its global prevalence has risen sharply in recent decades and is now estimated to affect approximately 25–30% of the adult population worldwide [3,4]. This rise reflects increasing rates of obesity and sedentary behavior and makes MetS one of the most pressing public health challenges of the twenty-first century.

Oral health, particularly periodontal health, has emerged as an important dimension of systemic health. Periodontitis is a chronic inflammatory disease driven by dysbiotic subgingival biofilms, leading to progressive destruction of tooth-supporting tissues and, if untreated, to tooth loss [5]. Beyond local consequences, periodontitis is marked by significant systemic dissemination of inflammatory mediators and microbial products, placing it at the intersection of oral and systemic medicine.

Over the past two decades, accumulating epidemiological and pathophysiological evidence has established a significant, bidirectional association between MetS and periodontal disease. Individuals with MetS have a higher prevalence and greater severity of periodontitis than metabolically healthy individuals [6,7]. Conversely, periodontal inflammation may contribute to systemic inflammation by releasing pro-inflammatory mediators such as tumor necrosis factor-α (TNF-α), interleukin-6 (IL-6), and C-reactive protein (CRP), thereby potentially contributing to insulin resistance and other components of MetS [8]. This cyclical relationship is biologically plausible: both conditions share chronic inflammation, oxidative stress, and altered immune responses as core pathogenic features.

In obesity, a central feature of MetS, dysfunctional adipose tissue acts as an active endocrine organ, releasing adipokines and cytokines that modulate periodontal tissue responses and impair host defense [9]. At the same time, periodontal pathogens and their endotoxins can enter the systemic circulation, influence metabolic pathways, and contribute to endothelial dysfunction [10]. These shared mechanisms underscore a close, clinically meaningful, bidirectional link between oral and metabolic health.

While recent narrative reviews have addressed aspects of the metabolic syndrome–oral disease relationship [11,12], existing publications have either examined multiple oral conditions superficially without in-depth mechanistic analysis [11] or focused on epidemiological and mechanistic associations without systematically addressing the oral implications of pharmacological agents used in metabolic syndrome management [12]. The present review advances the field in three key respects: (1) it provides a molecularly detailed synthesis of the shared pathophysiological mechanisms underpinning the MetS–periodontitis relationship; (2) it systematically examines the oral effects of medications commonly used in MetS management, including sodium–glucose cotransporter-2 (SGLT2) inhibitors and glucagon-like peptide-1 (GLP-1) receptor agonists—an aspect not previously synthesized in narrative reviews; and (3) it translates the synthesized evidence into operationalized interdisciplinary clinical recommendations, supported by a formal methodological quality assessment using the SANRA framework.

Several narrative reviews have addressed components of this relationship; notably, Syed et al. recently examined the links between oral diseases and metabolic syndrome, focusing on general mechanisms [11]. However, the present review extends this body of evidence by synthesizing more recent literature through March 2026, incorporating emerging evidence on pharmacological interactions, specifically the oral effects of SGLT2 inhibitors and GLP-1 receptor agonists, and providing an integrated clinical framework with explicit recommendations for interdisciplinary screening and patient management. These aspects have not been comprehensively addressed in prior reviews and represent the principal added value of the present manuscript.

Understanding this interrelationship has significant clinical and public health implications. It supports interdisciplinary collaboration between dental and medical professionals and highlights the potential benefits of integrating periodontal assessment into the management of patients with metabolic disorders. This review synthesizes current evidence on the bidirectional relationship between MetS and periodontitis, using the SANRA (Scale for the Assessment of Narrative Review Articles) framework [13], with a focus on shared pathophysiological mechanisms, epidemiological evidence, interventional outcomes, and interdisciplinary clinical implications.

## 2. Methods

This article is a narrative review conducted in accordance with the SANRA (Scale for the Assessment of Narrative Review Articles) framework [13]. Narrative reviews synthesize a broad body of literature to provide comprehensive, contextually interpreted overviews of a topic. This format was selected as appropriate given the scope and the heterogeneity of study designs in the available literature.

A literature search was conducted in PubMed, Scopus, and Web of Science for articles published between January 2021 and March 2026. The search strategy combined free-text and MeSH terms, including “metabolic syndrome”, “metabolic syndrome X”, “insulin resistance”, “dyslipidemia”, “oral health”, “periodontal disease”, “dental caries”, and “oral microbiota”, linked with Boolean operators (AND, OR).

Inclusion criteria:

- Original studies (cross-sectional, case–control, prospective cohort) and systematic reviews or meta-analyses;
- Studies investigating the association between MetS or its components and oral diseases or the oral microbiota;
- Full text available in English.

Exclusion criteria:

- Duplicate publications;
- Animal or in vitro studies, except those directly informative about mechanistic pathways;
- Conference abstracts without accessible full text.

Articles were selected in two stages: an initial screening of titles and abstracts by two independent reviewers, followed by a full-text review to confirm eligibility. Disagreements were resolved by consensus. Relevant data were extracted and synthesized narratively, organized by theme (pathophysiological mechanisms, epidemiological evidence, interventional data, and clinical implications). Reference lists of included articles were also screened to identify relevant studies not captured by the database search. No formal risk-of-bias assessment was performed, consistent with the narrative review format; however, the quality and study design of the cited evidence are noted throughout.

**Figure 1:**
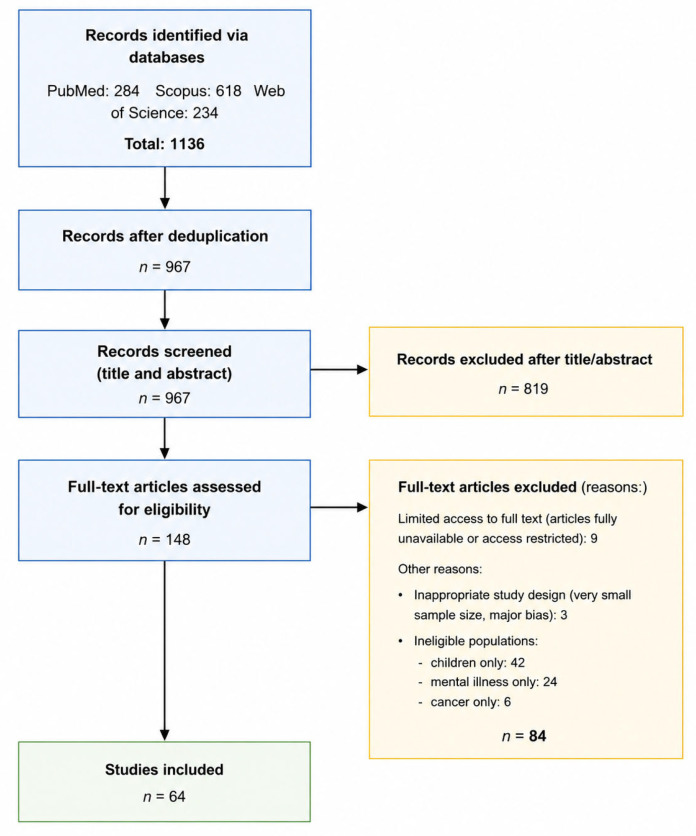
PRISMA flow diagram of literature search and study selection process.

The initial database search yielded 1136 records (PubMed: 284, Scopus: 618, Web of Science: 234). After deduplication, 967 records were screened by title and abstract, and 819 were excluded. Of the 148 full-text articles assessed for eligibility, 84 were excluded due to limited full-text access (*n* = 9), inappropriate study design (*n* = 3), or ineligible populations (studies restricted to pediatric, female-only, elderly, or pregnant populations: *n* = 42; mental illness only: *n* = 24; cancer only: *n* = 6). A total of 64 studies were included in the final narrative synthesis (Figure 1).

## 3. Results

### 3.1. Common Pathophysiological Mechanisms

**Figure 2:**
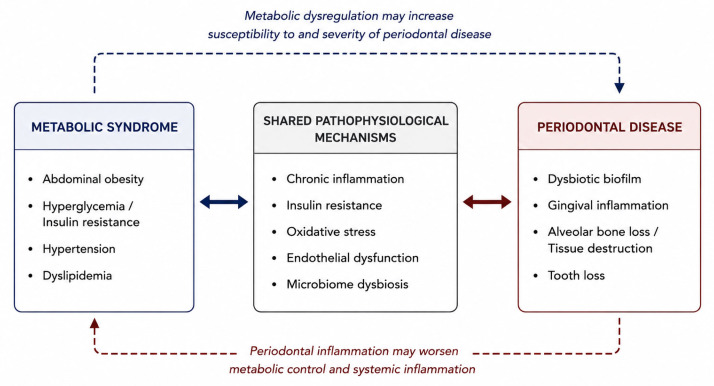
The bidirectional interrelationship between metabolic syndrome and oral health illustrates shared pathophysiological pathways, including systemic inflammation, insulin resistance, oxidative stress, adipokine dysregulation, endothelial dysfunction, and oral–gut microbiome interactions.

The bidirectional relationship between MetS and oral health is supported by several overlapping pathophysiological mechanisms, including chronic inflammation, insulin resistance, oxidative stress, adipokine imbalance, endothelial dysfunction, and microbiome interactions (Figure 2).

Figure 2 illustrates the principal bidirectional pathways linking MetS and oral health, highlighting the roles of systemic inflammation, insulin resistance, oxidative stress, adipokine dysregulation, endothelial dysfunction, and microbiome interactions. Each pathway is discussed in detail in the following subsections.

#### 3.1.1. Chronic Inflammation

Chronic low-grade inflammation is a defining feature of both MetS and periodontitis. In MetS, visceral adipose tissue functions as an active endocrine organ, secreting pro-inflammatory cytokines including TNF-α, IL-6, and resistin, while downregulating the protective adipokine adiponectin [14]. These mediators sustain systemic inflammation and promote insulin resistance.

In periodontitis, an exaggerated host inflammatory response to subgingival biofilms increases local and systemic levels of IL-1β, IL-6, TNF-α, and CRP [9]. Periodontal pathogens and their lipopolysaccharide endotoxins can translocate into the bloodstream, further amplifying systemic inflammation. Elevated circulating CRP in patients with periodontitis provides direct evidence of this systemic extension [15]. The resulting shared inflammatory profile creates a bidirectional feedback loop: systemic inflammation associated with MetS can exacerbate periodontal tissue destruction, while periodontal inflammation can worsen metabolic dysregulation.

#### 3.1.2. Insulin Resistance

Insulin resistance is a central pathogenic feature of MetS. Pro-inflammatory cytokines, particularly TNF-α, interfere with insulin signaling by impairing insulin receptor substrate (IRS) phosphorylation and reducing GLUT4 glucose transporter expression, thereby decreasing peripheral glucose uptake [16].

Periodontal inflammation may contribute to insulin resistance through similar mechanisms. Systemic dissemination of inflammatory mediators from periodontal tissues impairs insulin signaling in peripheral tissues [17]. Clinical interventional data support a causal link: periodontal therapy modestly improves glycemic control in patients with metabolic disorders [18]. Conversely, hyperglycemia promotes the formation of advanced glycation end-products (AGEs), which interact with their receptor (RAGE), thereby enhancing oxidative stress and inflammatory responses in periodontal tissues and accelerating tissue degradation [19].

#### 3.1.3. Oxidative Stress

Oxidative stress contributes to the pathogenesis of both MetS and periodontitis. In MetS, excess free fatty acids and hyperglycemia increase the production of reactive oxygen species (ROS), leading to endothelial dysfunction and cellular damage [20]. In periodontitis, activated neutrophils generate ROS as part of the antimicrobial host defense; however, excessive ROS can contribute to connective tissue degradation and alveolar bone resorption [21]. The cumulative oxidative burden from both conditions may accelerate tissue damage at local and systemic levels.

#### 3.1.4. Adipokines and Immune Modulation

Adipose tissue-derived mediators (adipokines) play a key role in linking MetS to periodontal inflammation. Leptin, resistin, and visfatin exert pro-inflammatory effects, whereas adiponectin exerts anti-inflammatory effects. In obesity and MetS, adiponectin levels are reduced, diminishing its protective effects on periodontal tissues [14]. These adipokine imbalances alter immune cell function, increase macrophage activation, and promote periodontal tissue degradation, potentially explaining the greater severity of periodontitis observed in patients with MetS [22].

#### 3.1.5. Endothelial Dysfunction

Endothelial dysfunction is a hallmark of MetS and a precursor to cardiovascular complications. Periodontal pathogens, notably Porphyromonas gingivalis, can invade endothelial cells and promote vascular inflammation [23]. Circulating bacterial components trigger endothelial activation, upregulate adhesion molecule expression, and contribute to atherogenesis. Oral inflammation may therefore exacerbate preexisting vascular abnormalities in patients with MetS, accelerating progression toward cardiovascular events.

#### 3.1.6. Oral Microbiome Interactions

**Figure 3:**
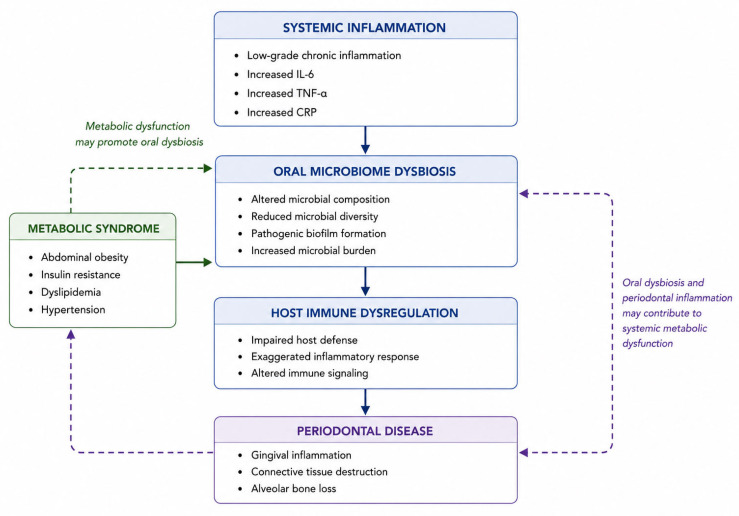
Proposed mechanistic model illustrating interactions between metabolic syndrome, oral microbiome dysbiosis, host immune dysregulation, and periodontal disease.

The oral microbiota comprises a diverse community of bacteria, fungi, and viruses that form biofilms and maintain local homeostasis. Dysbiosis of this community is implicated in caries and periodontitis [23,24]. Importantly, emerging evidence suggests that systemic metabolic changes can alter the oral microbial composition, while oral pathogens, particularly *P. gingivalis*, can influence the gut microbiota and systemic metabolism via an oro-intestinal axis [25] (Figure 3).

Animal models show that oral administration of *P. gingivalis* induces intestinal dysbiosis and insulin resistance, supporting the biological plausibility of microbiome-mediated metabolic disruption [25]. Human studies have reported distinct oral microbiome profiles in individuals with MetS compared with metabolically healthy controls, including enrichment of taxa associated with systemic inflammation [23,24]. This emerging field provides a mechanistic basis for future targeted therapeutic strategies.

The mechanisms summarized in Table 1 highlight the complex biological interplay between metabolic syndrome and periodontal disease.

### 3.2. Epidemiological Evidence

#### 3.2.1. Cross-Sectional Studies

A substantial body of cross-sectional studies consistently indicates that individuals with MetS have a higher prevalence and greater severity of periodontitis than metabolically healthy controls. Population-based studies show that components of MetS, including abdominal obesity, hyperglycemia, and dyslipidemia, are independently associated with increased periodontal probing depth and clinical attachment loss [6,7].

A systematic review and meta-analysis of observational studies found that subjects with MetS were significantly more likely to have periodontitis than those without MetS, even after adjusting for confounders such as age, smoking, and socioeconomic status, with a pooled odds ratio of approximately 1.7 [6,26,27,28].

**Figure 4:**
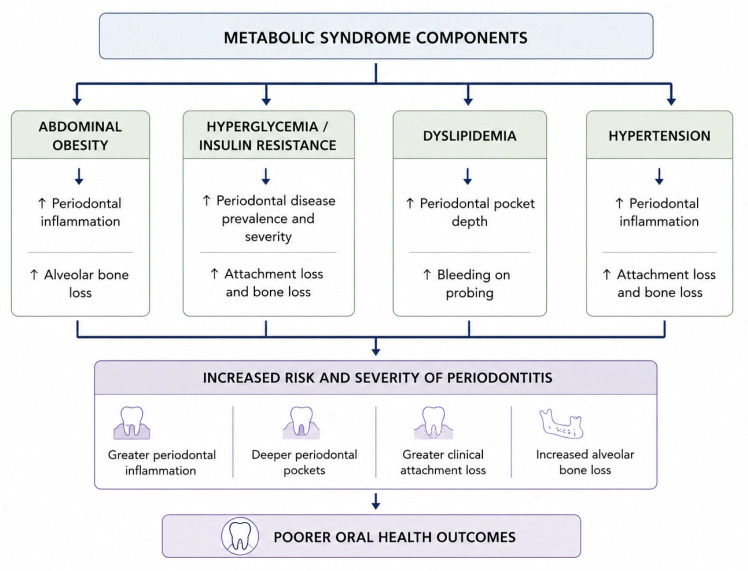
Association between individual components of metabolic syndrome (abdominal obesity, hyperglycemia, dyslipidemia, and hypertension) and periodontitis severity: summary of cross-sectional and longitudinal epidemiological evidence.

Abdominal obesity is indicated by increased waist circumference, a marker of visceral adiposity consistently linked to periodontal inflammation and alveolar bone loss [29]. Hyperglycemia and insulin resistance have been linked to poorer periodontal health, with individuals exhibiting elevated HbA1c levels showing greater periodontal deterioration than normoglycemic subjects, independent of other established risk factors [30]. Dyslipidemia, characterized by elevated total cholesterol and triglycerides and reduced HDL cholesterol concentrations, has been associated with greater periodontal pocket depth and increased bleeding on probing, suggesting a dose-dependent relationship between metabolic burden and periodontal disease severity [31,32,33,34]. Hypertension has also been associated with poorer periodontal and dental status in large epidemiological cohorts, independent of antihypertensive medication use [35]. These findings support the concept that MetS components may act synergistically to increase susceptibility to periodontal inflammation (Figure 4).

#### 3.2.2. Longitudinal Studies

Longitudinal data are essential to infer temporal relationships and disease progression. Cohort studies indicate that individuals with MetS at baseline are at higher risk of developing periodontitis or experiencing more rapid periodontal deterioration over time. Longitudinal analyses of adult cohorts have shown that MetS independently predicts worsening periodontal parameters, including attachment loss and tooth loss, after several years of follow-up [36,37].

Emerging evidence also supports the reverse direction: periodontal disease at baseline may increase the risk of developing metabolic disorders. In prospective studies, moderate-to-severe periodontitis has been associated with an increased incidence of insulin resistance and new-onset components of MetS, supporting the hypothesis that poor oral health may precede systemic metabolic changes [7,38]. A Finnish cohort study published in 2026 provided particularly robust evidence for this bidirectionality after controlling for a comprehensive range of confounders [26].

### 3.3. Interventional Evidence

#### 3.3.1. Effects of Periodontal Therapy on Metabolic Parameters

Meta-analyses of interventional studies suggest that non-surgical periodontal therapy, primarily scaling and root planning (SRP), yields modest but statistically significant improvements in glycemic control, as measured by reductions in HbA1c, among individuals with T2DM and prediabetes [18,39]. These reductions are clinically meaningful, particularly in the context of multifactorial MetS management.

Beyond glycemic control, some studies report favorable changes in systemic inflammatory markers, including CRP and IL-6, after periodontal treatment, which may improve insulin sensitivity and reduce cardiovascular risk [8,40]. A 2025 systematic review specifically examining MetS parameters found that periodontal therapy produced measurable improvements across multiple MetS components, though the effect sizes were modest and heterogeneity was considerable [39].

#### 3.3.2. Combined Medical and Dental Interventions

Integrated care models that combine periodontal therapy with lifestyle modification and/or metabolic pharmacotherapy may yield synergistic effects beyond those of either intervention alone. Although larger, long-term randomized controlled trials (RCTs) are needed, available evidence from pilot studies suggests that multidisciplinary interventions in individuals with MetS and periodontitis are associated with greater improvements in both periodontal status and metabolic biomarkers [41,42].

### 3.4. MetS-Associated Comorbidities: T2DM and Cardiovascular Disease

#### 3.4.1. Type 2 Diabetes Mellitus

Individuals with T2DM and coexisting MetS constitute a particularly high-risk group for severe periodontitis. Clinical studies show that the coexistence of MetS exacerbates periodontal damage in patients with T2DM beyond the effect of either condition alone [43]. Chronic periodontal infection releases pro-inflammatory mediators, including TNF-α, into the systemic circulation, complicating glycemic management [44]. This creates a self-reinforcing cycle in which metabolic dysregulation worsens periodontal status, and vice versa.

#### 3.4.2. Cardiovascular Diseases

Patients with cardiovascular disease and coexisting MetS also show increased susceptibility to periodontal inflammation, reflecting shared inflammatory and vascular pathways linking oral and systemic vascular health [45,46]. Individuals with periodontitis face an elevated risk of cardiovascular events, including myocardial infarction, heart failure, atrial fibrillation, and stroke [46]. Periodontitis-associated systemic inflammation, mediated by elevated circulating cytokines, CRP, and neutrophil extracellular traps (NETs), contributes to atherosclerosis and increases vulnerability to plaque rupture [47]. Certain periodontal pathogens, notably *P. gingivalis*, have been identified within atherosclerotic vessels, providing direct microbiological evidence of a mechanistic link [23]. A summary of landmark and recent studies supporting these associations is presented in Table 2.

### 3.5. Emerging Digital and AI-Assisted Tools in Interdisciplinary Screening

Against the backdrop of the interdisciplinary care framework advocated throughout this review, integrating artificial intelligence (AI) and machine learning into clinical dentistry and medicine is an emerging avenue with specific relevance to the bidirectional screening and coordinated management of patients with concurrent metabolic syndrome and periodontal disease. AI-assisted tools are increasingly evaluated for automated radiographic analysis, periodontal grading, caries detection, and risk stratification, with several models demonstrating diagnostic accuracy comparable to that of experienced clinicians in controlled settings [48]. In the context of MetS and oral health, AI-based decision support systems could theoretically facilitate bidirectional screening by flagging high-risk patients for referral across dental and medical settings and by integrating metabolic and periodontal risk indicators within shared electronic health record platforms. However, significant challenges remain, including the need for prospective clinical validation, algorithmic transparency, data standardization across health systems, and careful consideration of equity and access. D’Albis and Capodiferro [48] have recently highlighted both the promise and the prudence required when implementing AI in oral surgery practice, emphasizing that current AI tools should be regarded as decision-support aids rather than autonomous diagnostic systems [48]. These considerations apply equally to the broader interdisciplinary screening context discussed in the present review, and future research should explicitly address the clinical validation and implementation challenges of AI-assisted tools in this field. Developing validated AI-assisted screening protocols that integrate periodontal and metabolic risk indicators represents a promising direction for operationalizing the interdisciplinary care pathways described in Section 4 of this review.

## 4. Discussion

The evidence synthesized in this review supports a robust, bidirectional relationship between MetS and oral health, predominantly mediated by shared inflammatory, metabolic, and microbial pathways. Chronic systemic inflammation, immune dysregulation, oxidative stress, and adipokine imbalances are implicated in both periodontal pathogenesis and metabolic dysregulation [9]. Periodontal pathogens and their products disseminate systemically, contributing to endothelial dysfunction, insulin resistance, and metabolic inflammation, key features of MetS. Conversely, the metabolic milieu of MetS, characterized by hyperglycemia, dyslipidemia, and adipose-driven inflammation, creates an environment that predisposes individuals to more severe and rapidly progressing periodontitis. Importantly, while the biological plausibility of this bidirectional relationship is well-established, the causal directionality remains difficult to disentangle from current observational data. Most cross-sectional studies cannot determine temporal precedence, and residual confounding by smoking, socioeconomic status, and dietary patterns remains a concern even in well-adjusted longitudinal analyses.

### 4.1. Implications for Risk Assessment and Early Detection

#### 4.1.1. Periodontal Screening in Patients with MetS

Patients diagnosed with MetS should be considered at elevated risk of periodontitis. Given this association, including periodontal assessment in the routine medical evaluation of patients with obesity, hypertension, dyslipidemia, or impaired glucose tolerance may enable earlier detection and management of oral inflammation, with potential downstream systemic benefits [6,43].

Oral examinations for patients with MetS should be systematic and go beyond routine dental assessment. Clinicians are encouraged to evaluate key periodontal parameters, including bleeding on probing (BOP), probing pocket depth (PPD), clinical attachment level (CAL), tooth mobility, and radiographic alveolar bone loss, as well as salivary flow rate, signs of xerostomia, and oral mucosal lesions, all of which are more prevalent in metabolically compromised patients. Referral to medical specialists should be considered when severe or generalized periodontitis is identified alongside systemic risk indicators such as abdominal obesity, hypertension, or a family history of diabetes, in accordance with the EFP–IDF collaborative guidelines [10].

#### 4.1.2. Metabolic Screening in Dental Settings

Dental professionals are well positioned to identify patients at risk for metabolic disorders. Clinical signs such as severe periodontitis, recurrent periodontal abscesses, xerostomia, or impaired wound healing may indicate underlying metabolic abnormalities. Screening measures available in a dental setting, including blood pressure measurement, body mass index (BMI) assessment, waist circumference measurement, and referral for fasting glucose or HbA1c testing, can facilitate early detection of MetS or prediabetes [19]. This bidirectional screening approach supports preventive healthcare and may reduce long-term systemic complications.

### 4.2. Impact of Periodontal Therapy on Metabolic Outcomes

#### 4.2.1. Glycemic Control

Interventional studies indicate that non-surgical periodontal therapy (SRP) can achieve modest but clinically meaningful reductions in HbA1c in patients with diabetes and metabolic dysregulation, with meta-analyses reporting improvements at 3–4 months of follow-up [18,39]. Reductions in systemic inflammatory markers (CRP, IL-6) following periodontal treatment may indirectly reduce insulin resistance and cardiovascular risk [15,40].

#### 4.2.2. Systemic Inflammation and Cardiovascular Risk

Periodontal treatment has been associated with improvements in endothelial function and significant reductions in inflammatory biomarkers [40]. Such findings suggest that periodontal management may confer broader cardiovascular benefits in patients with MetS. However, these conclusions must be interpreted with caution: available trials are generally short-term, underpowered, and heterogeneous in their periodontal and metabolic endpoints. Large RCTs with hard cardiovascular outcomes and standardized periodontal protocols remain an unmet research priority.

### 4.3. Implications for Treatment Planning

#### 4.3.1. Modified Treatment Protocols

Patients with MetS frequently present with comorbidities, hypertension, and altered glucose metabolism, which influence dental management. Preoperative monitoring of blood pressure and glycemic control, stress-reduction strategies, and tailored maintenance intervals are advisable before invasive procedures. Obesity and systemic inflammation may also impair wound healing and increase susceptibility to postoperative infections, necessitating rigorous infection control and more frequent supportive periodontal care.

#### 4.3.2. Pharmacological Considerations

Medications commonly prescribed for MetS management have oral implications that clinicians should recognize. Calcium channel blockers (e.g., amlodipine, nifedipine) may cause gingival hyperplasia, and antihypertensive and antidiabetic agents are among the most common causes of drug-induced xerostomia, increasing caries risk [49]. Statins have been proposed to exert anti-inflammatory and potentially periodontal-protective effects, representing a possible pharmacological avenue for adjunctive periodontal management [33,50].

Sodium–glucose cotransporter-2 inhibitors (SGLT2is), increasingly prescribed for T2DM and heart failure, may increase glucose concentrations in saliva and gingival crevicular fluid, potentially altering the oral microbiome, compromising mucosal integrity, and causing burning sensations, glossitis, or tongue edema [51]. Evidence for these effects is currently limited to case reports and small series, and clinicians should monitor patients receiving SGLT2is for oral mucosal changes.

GLP-1 receptor agonists, widely used to treat T2DM and obesity, may impair oral health through gastrointestinal side effects (nausea, vomiting, acid reflux), which can cause enamel erosion, as well as reduced food intake and xerostomia, collectively increasing the risk of dental caries, gingival disease, and tooth sensitivity [52]. Conversely, emerging evidence from in vitro studies and scoping reviews suggests a potential beneficial role for GLP-1 receptor agonists and DPP-4 inhibitors in reducing periodontal inflammation [52], although clinical confirmation is required. Clinicians should counsel patients receiving these agents on oral hygiene vigilance and regular dental review. The principal oral manifestations associated with medications commonly used to manage metabolic syndrome are summarized in Table 3.

#### 4.3.3. Lifestyle and Preventive Counseling

Lifestyle factors—poor diet, physical inactivity, tobacco use, and excess alcohol consumption—are shared risk factors for both MetS and periodontal disease [53,54,55,56]. Dental professionals can contribute to lifestyle modification counseling, including guidance on reducing refined sugar intake and adopting anti-inflammatory dietary patterns, support for smoking cessation, and encouragement of physical activity [57]. Addressing these common determinants through a shared-risk-factor approach has the potential to simultaneously improve oral and metabolic health [58].

#### 4.3.4. Interdisciplinary Collaboration

**Figure 5:**
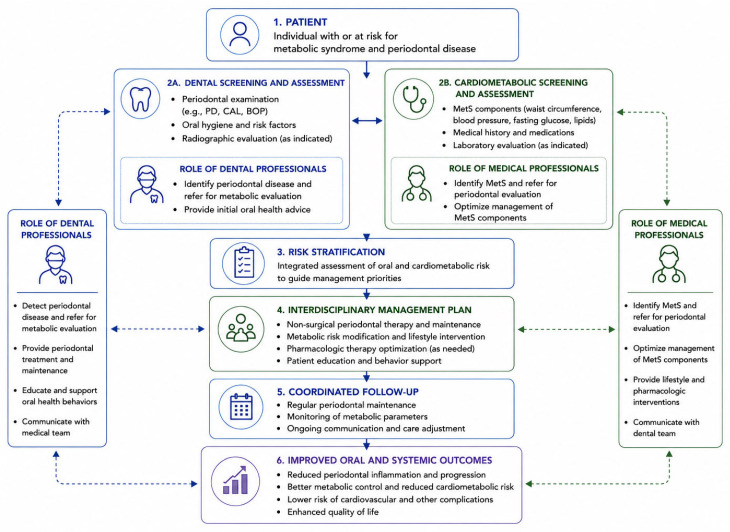
Proposed interdisciplinary care pathway for patients with concurrent metabolic syndrome and periodontal disease.

The bidirectional interrelationship between MetS and oral health underscores the need for integrated models of care [59]. Collaboration among dentists, endocrinologists, cardiologists, and general practitioners can improve patient outcomes. Shared electronic health records, established referral pathways, and collaborative management protocols facilitate comprehensive, patient-centered care [60,61]. Consensus statements from periodontal and diabetes professional organizations emphasize the importance of interdisciplinary communication in managing patients with co-existing metabolic and periodontal diseases [10] (Figure 5).

Figure 5 depicts a proposed integrated care model for patients with concurrent metabolic syndrome and periodontal disease, highlighting the key roles of dental and medical professionals in coordinating screening, treatment, and long-term follow-up.

### 4.4. Public Health Implications

At the population level, recognizing oral health as integral to systemic health can inform public health policy. Preventive dental programs targeting high-risk populations, including individuals with obesity, prediabetes, or established MetS, could help reduce the prevalence of both oral and metabolic diseases [62,63]. Integrating oral health into chronic disease prevention strategies aligns with global initiatives that promote holistic, preventive approaches to non-communicable disease management [64]. Emerging AI-assisted decision support tools, discussed in Section 3.5, may further support population-level screening efforts by enabling more efficient identification of high-risk individuals across both dental and medical settings.

### 4.5. Limitations

This review has several limitations that should be acknowledged. As a narrative review, it did not use a pre-registered systematic protocol for synthesizing the literature, and the study selection, while broad, may be subject to selection bias. Most included epidemiological studies are cross-sectional, precluding definitive causal inference. Heterogeneity in MetS definitions, periodontitis classification systems, and population characteristics across studies limits direct comparability. No formal quality appraisal tool was applied to individual studies. The review focused primarily on periodontitis; associations between MetS and other oral conditions (caries, oral mucosal disease, xerostomia) are less thoroughly represented in the current literature and warrant further investigation.

The oral microbiome has emerged as a potential therapeutic target linking metabolic syndrome and periodontal disease. Growing interest in microbiome modulation through dietary interventions, probiotics, and personalized preventive strategies reflects a shift toward more integrated approaches to chronic disease management. While evidence regarding clinical efficacy remains limited, future research may help determine whether microbiome-based interventions can complement conventional periodontal and metabolic therapies.

### 4.6. Recommendations for Future Research

Several important gaps in the current evidence base warrant targeted future investigation. First, large-scale randomized controlled trials with standardized case definitions of both metabolic syndrome and periodontitis, long follow-up periods, and hard clinical endpoints, such as cardiovascular events and T2DM incidence, are needed to establish causal directionality and quantify the systemic benefit of periodontal intervention. Second, prospective cohort studies should examine whether early periodontal treatment can delay or prevent the onset of full MetS in at-risk populations. Third, the oral microbiome represents a promising yet underexplored therapeutic target, but mechanistic studies are needed to characterize specific dysbiotic signatures associated with components of MetS and to evaluate probiotic or prebiotic interventions. Fourth, validated interdisciplinary care pathways and referral protocols that integrate dental and medical risk assessment should be developed and tested across diverse healthcare settings. Fifth, future research should assess the cost-effectiveness of integrated periodontal and metabolic screening programs to support implementation in routine clinical practice.

Emerging digital and artificial intelligence (AI)-assisted tools represent an additional avenue for future development in this field. AI-driven decision support systems have the potential to enhance interdisciplinary risk stratification by integrating periodontal and metabolic parameters, flagging high-risk patients for cross-disciplinary referral, and supporting personalized treatment planning. However, their application in clinical dentistry and metabolic medicine remains nascent, and evidence for their real-world validity, equity, and cost-effectiveness is limited [48]. Rigorous evaluation of AI-assisted screening tools in the context of MetS–periodontitis management should be a priority in the future research agenda.

Sixth, future studies should prioritize the standardization of diagnostic criteria for both metabolic syndrome and oral diseases, as the current heterogeneity in case definitions across studies substantially limits comparability and the strength of pooled estimates. Seventh, research should extend beyond periodontitis to encompass the full spectrum of oral health outcomes associated with metabolic dysfunction, including dental caries, tooth loss, alterations in salivary flow, xerostomia, and oral mucosal lesions, which remain insufficiently characterized in the context of MetS. Finally, multicentric studies that include underrepresented populations, particularly from Eastern Europe, sub-Saharan Africa, and Latin America, are needed to assess whether the observed associations are generalizable across diverse ethnic and socioeconomic contexts.

## 5. Conclusions

The bidirectional relationship between MetS and periodontitis is supported by converging pathophysiological, microbiological, and epidemiological evidence. Chronic low-grade inflammation is a common mechanistic thread that may sustain this bidirectional relationship and has broad clinical consequences for both conditions. Non-surgical periodontal therapy appears to yield modest but consistent improvements in glycemic control and systemic inflammatory markers, suggesting tangible systemic benefits beyond the oral cavity. Integrating periodontal assessment into MetS management and screening for metabolic risk factors in dental settings is a pragmatic, evidence-informed proposal for interdisciplinary preventive medicine, pending confirmation by large-scale RCTs with standardized endpoints. Future research priorities include large-scale RCTs examining the long-term impact of periodontal therapy on hard MetS endpoints beyond HbA1c; prospective cohort studies using standardized definitions of MetS and periodontitis; investigation of oral microbiome-targeted interventions; and the development of validated interdisciplinary care pathways. Population-wide preventive education emphasizing oral hygiene, healthy diet, weight management, and regular dental visits may help reduce the burden of both conditions simultaneously. Recognition of oral health as a core component of cardiometabolic health may facilitate earlier risk identification, more effective prevention strategies, and improved long-term outcomes through interdisciplinary care.

## Figures and Tables

**Table 1:** Shared pathophysiological mechanisms linking metabolic syndrome and periodontal disease.

Mechanism	Key Mediators/Biomarkers	Role in Metabolic Syndrome	Role in Periodontitis	Clinical Consequences
Chronic inflammation	TNF-α, IL-6, CRP	Elevated TNF-α, IL-6, and CRP contribute to systemic inflammatory burden.	Promotes periodontal tissue destruction and alveolar bone resorption.	Insulin resistance and increased cardiometabolic risk.
Oxidative stress	ROS, AGEs, RAGE	ROS overproduction contributes to endothelial and metabolic dysfunction.	ROS-mediated connective tissue degradation and inflammatory damage.	Accelerated vascular and periodontal deterioration.
Insulin resistance	HbA1c, fasting glucose, HOMA-IR	Impaired glucose metabolism and altered insulin signaling.	Impaired periodontal healing and exaggerated inflammatory response.	Poor glycemic control and increased periodontal severity.
Adipokine imbalance	Leptin, adiponectin, resistin	Reduced adiponectin and increased leptin/resistin levels.	Enhanced inflammatory response in periodontal tissues.	Greater susceptibility to severe periodontitis.
Endothelial dysfunction	ICAM-1, VCAM-1, nitric oxide dysregulation	Vascular inflammation and impaired microcirculation.	Reduced periodontal tissue perfusion and healing.	Higher cardiovascular risk.
Microbiome dysbiosis	Porphyromonas gingivalis, altered oral microbiota	Altered oral and gut microbial composition.	Pathogenic biofilm formation and chronic inflammation.	Systemic metabolic disruption and oral–gut axis alterations.

TNF-α, tumor necrosis factor-alpha; IL-6, interleukin-6; CRP, C-reactive protein; ROS, reactive oxygen species; AGEs, advanced glycation end-products; RAGE, receptor for advanced glycation end-products; HbA1c, glycated hemoglobin; HOMA-IR, homeostatic model assessment of insulin resistance; ICAM-1, intercellular adhesion molecule-1; VCAM-1, vascular cell adhesion molecule-1.

**Table 2:** Summary of key studies included in review.

Author, Year [Ref.]	Study Design	Population	Key Finding	Clinical Relevance
Campos et al., 2022 [6]	Systematic review & meta-analysis	Multiple populations	Significant association between MetS components and periodontitis; OR 1.71 for MetS vs. controls	Supports routine periodontal screening in individuals with MetS.
Santoso et al., 2022 [20]	Cross-sectional (national survey)	Indonesian adults (*n* = 30,000+)	MetS components independently associated with higher periodontitis prevalence	Suggests that individual MetS components contribute independently to periodontal risk.
Kotin et al., 2022 [27]	Cross-sectional (Hamburg City Health Study)	German adults (*n* = 10,000+)	Periodontitis significantly associated with MetS after adjusting for confounders	Supports the association beyond major confounding factors.
Rosário-Dos-Santos et al., 2023 [42]	Systematic review & meta-analysis	Multiple populations	Severe periodontitis associated with higher odds of MetS (OR 1.86)	Strengthens evidence for a robust MetS–periodontitis relationship.
Saito et al., 2024 [36]	Longitudinal cohort (8-year)	Japanese adults	Periodontitis predicted incident metabolic syndrome (HR 1.44)	Indicates that periodontitis may serve as an early marker of future metabolic dysfunction.
Kinnunen et al., 2026 [26]	Longitudinal cohort	Finnish adults	Bidirectional association: periodontitis predicted MetS and vice versa	Provides strong support for the bidirectional relationship between oral and metabolic health.
Doke et al., 2021 [18]	Randomized controlled trial	Adults with MetS	Dental intervention improved MetS parameters including waist circumference and HbA1c	Suggests that periodontal therapy may contribute to metabolic improvement.
Simonelli et al., 2025 [39]	Systematic review	Adults with MetS	Periodontal treatment produced modest but significant reductions in MetS parameters	Supports integration of periodontal care into comprehensive MetS management.
Ayuthaya et al., 2025 [7]	Prospective cohort (10-year)	Thai adults	Periodontal disease at baseline predicted development of MetS components	Suggests that poor periodontal health may precede metabolic deterioration.
Li et al., 2025 [28]	Cross-sectional + cohort	Chinese population	Bidirectional association confirmed; periodontitis severity correlated with MetS score	Supports the consistency of the association across study designs and populations.

MetS: metabolic syndrome; OR: odds ratio; HR: hazard ratio; HbA1c: glycated hemoglobin. The selection represents landmark and recent studies; the complete list of cited evidence is provided in the References Section.

**Table 3:** Oral implications of medications commonly used in metabolic syndrome management.

Medication Class	Examples	Oral Manifestations	Potential Dental Implications
Calcium channel blockers	Amlodipine, nifedipine	Gingival hyperplasia	Increased periodontal inflammation and plaque retention.
Antihypertensive agents	Various classes	Xerostomia	Increased caries risk and oral discomfort.
SGLT2 inhibitors	Empagliflozin, dapagliflozin	Burning mouth, xerostomia, mucosal alterations	Potential changes in oral microbiota and mucosal integrity.
GLP-1 receptor agonists	Semaglutide, liraglutide	Reflux-associated enamel erosion, xerostomia	Tooth sensitivity and increased caries susceptibility.
Statins	Atorvastatin, rosuvastatin	Potential anti-inflammatory effects	Possible adjunctive periodontal benefits.

SGLT2: sodium–glucose cotransporter-2; GLP-1: glucagon-like peptide-1.

## Data Availability

No new data were created or analyzed in this study.

## Abbreviations

AGEs	advanced glycation end-products
BMI	body mass index
CRP	C-reactive protein
CVD	cardiovascular disease
DPP-4	dipeptidyl peptidase-4
GLP-1	glucagon-like peptide-1
GLUT4	glucose transporter type 4
HbA1c	glycated hemoglobin
HR	hazard ratio
IDF	International Diabetes Federation
IL-1β	interleukin-1 beta
IL-6	interleukin-6
IRS	insulin receptor substrate
MetS	metabolic syndrome
NETs	neutrophil extracellular traps
OR	odds ratio
RAGE	receptor for advanced glycation end-products
RCTs	randomized controlled trials
ROS	reactive oxygen species
SANRA	Scale for the Assessment of Narrative Review Articles
SGLT2i	sodium–glucose cotransporter-2 inhibitors
SRP	scaling and root planning
T2DM	type 2 diabetes mellitus
TNF-α	tumor necrosis factor-alpha
